# *Trapa bispinosa* Roxb. extract lowers advanced glycation end-products and increases live births in older patients with assisted reproductive technology: a randomized controlled trial

**DOI:** 10.1186/s12958-021-00832-y

**Published:** 2021-09-27

**Authors:** Masao Jinno, Ryoji Nagai, Masayoshi Takeuchi, Aiko Watanabe, Koji Teruya, Hikari Sugawa, Naohisa Hatakeyama, Yuichi Jinno

**Affiliations:** 1 Women’s Clinic Jinno, 3-11-7 Kokuryou-chou, Choufu City, Tokyo 182-0022 Japan; 2 Laboratory of Food and Regulation Biology, Graduate School of Agriculture, Tokai University, Kumamoto, 862-8652 Japan; 3 Department of Advanced Medicine, Medical Research Institute, Kanazawa Medical University, Ishikawa, 920-0293 Japan; 4 Faculty of Health Sciences, Kyorin University, Mitaka City, Tokyo 181-8612 Japan

**Keywords:** Advanced glycation end-products (AGE), Assisted reproductive technology, *Trapa bispinosa* Roxb., Live birth rate, oocyte developmental competence, endometrial receptivity, Water chestnut

## Abstract

**Background:**

Advanced glycation end-products (AGE), which accumulate with insulin resistance and aging, impair folliculogenesis and may decrease endometrial receptivity. Hishi (*Trapa bispinosa* Roxb.) extract, a safe herbal medicine, strongly inhibits AGE formation *in vitro*. We determined whether Hishi lowers AGE and increases live births in older assisted reproductive technology (ART) patients.

**Methods:**

This prospective randomized open-label controlled trial included 64 patients 38 to 42 years old undergoing ART with or without Hishi extract between June 11, 2015 and July 12, 2019. None had over 2 ART failures, diabetes, uterine anomalies, or exhausted ovarian reserve. After allocation, the Hishi group received Hishi extract (100 mg/day) until late pregnancy or failure. The control group received no extract. Both groups underwent 1 cycle of conventional infertility treatment; 1 long-protocol cycle of ovarian stimulation, oocyte retrieval, *in vitro* fertilization/intracytoplasmic sperm injection, and fresh embryo transfer (ET); and, if needed, cryopreserved ET until live birth or embryo depletion. Serum AGE were measured before and during ART, as were AGE in follicular fluid (FF).

**Results:**

Cumulative live birth rate among 32 Hishi patients was 47%, significantly higher than 16% among 31 controls (p<0.01; RR, 4.6; 95% CI, 1.4 – 15.0; 1 control dropped out). Live birth rate per ET, including fresh and cryopreserved, was significantly higher with Hishi (28% in 47 ET *vs.* 10% in 49 ET; p<0.05; RR, 3.4; 95% CI, 1.1-10.4). Among variables including age, day-3 FSH, anti-Müllerian hormone, and Hishi, logistic regression identified only Hishi as significantly associated with increased cumulative live birth (p<0.05; OR, 5.1; 95% CI, 1.4 - 18.3). Hishi significantly enhanced oocyte developmental potential, improved endometrial receptivity in natural cycles, and decreased AGE in serum and FF. Larger serum AGE decreases with Hishi were associated with more oocytes becoming day-2 embryos.

**Conclusions:**

Hishi decreased AGE in serum and FF and improved oocyte developmental potential and endometrial receptivity, increasing live births in older patients. Treatment of infertility by AGE reduction represents a new addition to infertility treatment. Therapeutic trials of Hishi for other AGE-associated diseases might be considered.

**Trial registration:**

UMIN registration in Japan (UMIN000017758) on June 1, 2015. https://www.umin.ac.jp/ctr/index.htm

## Background

Advanced glycation end-products (AGE) are cross-linked molecules formed by nonenzymatic reactions of carbonyl groups with amino groups of proteins, lipids, or nucleic acids [1]. AGE participate in aging [2] and in conditions involving insulin resistance (IR) such as metabolic syndrome, type 2 diabetes, hypertension, atherosclerosis, dyslipidemia, polycystic ovary syndrome (PCOS), and central obesity [1, 3, 4]. AGE toxicity results directly from macromolecular trapping and cross-linking, and indirectly from binding to AGE receptors (RAGE) [1, 5]. IR increases oxidative stress, promoting AGE formation; in turn, AGE induce inflammation, oxidative stress, and IR in vicious cycles escalating disease processes [1, 6]. Agents that decrease AGE such as aminoguanidine have shown benefits in animal and clinical studies involving diabetes, but clinical applicability is limited by long-term toxicity [1, 5].

AGE are linked to causes of infertility including PCOS, ovarian dysfunction, diminished ovarian reserve, ovarian aging, endometriosis, and male infertility [7–10]. Metformin, an insulin sensitizer, improves pregnancy rates in PCOS [11] and non-PCOS patients with repeated past failures of assisted reproductive technology (ART) [12]. Pathophysiology of infertility resembles that of other conditions involving IR.

The water chestnut (*Trapa bispinosa* Roxb.), a common aquatic plant in Asia, Europe, and Africa, has been consumed safely as food and herbal medicine for over 2000 years [13]. Pharmacologic investigations have demonstrated its antidiabetic, analgesic, antibiotic, and immunomodulatory activities [13]. An extract of its dried peel (Hishi extract) used as a cosmetic supplement was found to inhibit AGE formation *in vitro* [14].

We preliminarily examined adjunctive Hishi administration to older women with repeated ART attempts (mean age, 42.2 years), finding decreased AGE and an increased delivery rate of 19% [15]. We now undertook the present prospective randomized study to more conclusively determine whether Hishi extract improves ART outcomes by reducing AGE. Study patients, ranging from 38 to 42 years old, were likely to have age-related AGE accumulation. In Japan, 40.7% of ART treatments involve this age group, showing a delivery rate of only 9.3% [16]. Such patients risk becoming too old to overcome infertility.

## Materials and methods

### Study design

**Fig. 1:**
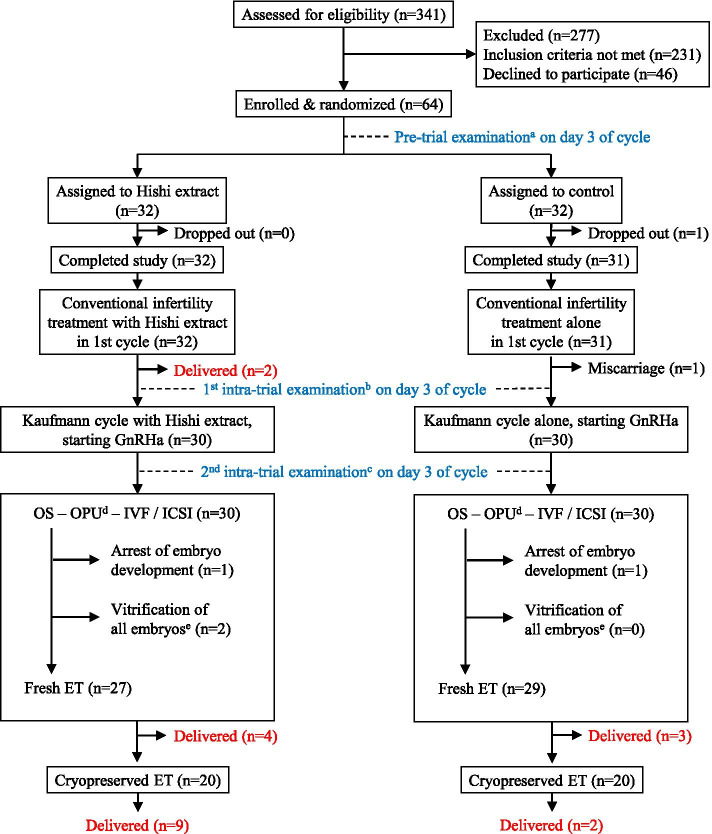
Participant flow diagram. ^a^ Serum AGE, biochemical parameters, oGTT values, and hormone concentrations were determined. ^b^ Hormones were determined. ^c^ Serum AGE, biochemical parameters, and oGTT values were determined. ^d^ Follicular fluid AGE were measured. ^e^ All embryos cryopreserved and no embryos transferred, to prevent ovarian hyperstimulation syndrome. OS, ovarian stimulation; OPU, oocyte pick-up; ET, embryo transfer

A prospective open-label randomized clinical trial was conducted to determine whether Hishi extract improved ART outcomes in patients at relatively advanced ages, and to clarify whether improvement was associated with AGE reduction. Between June 11, 2015 and July 12, 2019, a total of 341 patients presented to Women’s Clinic Jinno seeking ART treatment. Among them, 110 met study inclusion criteria. Forty-six declined participation, leaving 64 ART patients enrolled in the study and prospectively assigned to groups undergoing standard ART with or without concomitant Hishi extract administration. Randomization involved patients drawing from a box containing assignments in sealed envelopes mixed 1:1 (32 patients per group; Fig. 1). Neither patients nor investigators were blinded to resulting group assignments.

Inclusion criteria were age between 38 and 42 years, 2 or fewer previous ART failures, and a medical history free of diabetes mellitus, anti-diabetic medication, hyperlipidemia, hyperuricemia, congenital or acquired uterine infertility, severely diminished ovarian reserve (day-3 FSH 15 IU/L or greater, anti-Müllerian hormone [AMH] below 0.01 ng/mL, or absence of ovaries detectable by transvaginal ultrasonography), or a male partner with azoospermia.

On cycle day 3, enrolled patients underwent serum AGE measurements and clinical determinations including body mass index (BMI), systolic and diastolic blood pressure (SBP, DBP), 75-g oral glucose tolerance test (OGTT), routine biochemical analyses, and hormone measurements (pre-trial examination in Fig. 1). Daily oral administration of 100 mg of Hishi extract (Pregnasupport; Hayashikane Sangyo, Shimonoseki, Japan) then was initiated in the Hishi group only. No placebo was given to control subjects. To produce the Hishi extract, the dried and crashed rind of water chestnut (*Trapa bispinosa* Roxb.) was heated with water. Dextrin was added to the extracted solution before spray-drying to yield a powder.

In the initial menstrual cycle of the trial, both groups followed conventional infertility measures such as sexual intercourse or intrauterine insemination, with or without ovarian stimulation by clomiphene citrate or a recombinant FSH regimen. If patients did not become pregnant, hormone measurements were repeated on day 3 of the following cycle (first intra-trial examination in Fig. 1), immediately followed by a Kaufmann cycle consisting of sequential administration of conjugated estrogens (Premarin; Pfizer, Tokyo, Japan) for 7 days and estrogen-progestogen contraceptive pills (Planovar, Asuka Pharmaceuticals, Tokyo, Japan) for 14 days. On day 18 of the Kaufmann cycle, administration of a gonadotropin-releasing hormone (GnRH) agonist was initiated in preparation for long-protocol ART in the subsequent cycle.

On day 3 of the ART cycle, serum AGE measurements and clinical laboratory examinations were repeated, excluding hormone measurements (second intra-trial examination in Fig. 1). Follicular development then was stimulated by gonadotropin administration. Patients underwent transvaginal oocyte retrieval and *in vitro* fertilization (IVF) and/or intracytoplasmic sperm injection (ICSI). At oocyte retrieval, follicular fluid (FF) from follicles 18 mm or greater in diameter was retrieved and pooled. Specimens of serum and FF were frozen at -70°C and assayed later for AGE. Embryos were transferred 2, 3, or 5 days after IVF/ICSI. Remaining embryos, or all embryos if ovarian hyperstimulation syndrome (OHSS) was anticipated, were cryopreserved at the blastocyst stage using a vitrification method.

When IVF/ICSI and fresh embryo transfer (IVF/ICSI-fresh ET) failed to result in delivery, cryopreserved embryos were thawed and transferred (cryopreserved ET) in subsequent spontaneous cycles with and without administration of Hishi extract according to group assignment. Pregnancy was diagnosed by observation of a gestational sac. Miscarriage and delivery respectively were defined as pregnancy loss before 22 weeks and birth after 22 weeks. When successful pregnancy was achieved, Hishi usually was continued beyond 28 gestational weeks and then terminated. When all ET resulted in no pregnancy or in miscarriage, Hishi was discontinued.

When we designed and registered our clinical trial, we planned to use the number of superior embryos as the primary outcome measure. With a power of 0.80 and an α error of 0.05, we estimated the minimum number of participants needed to identify a difference between hypothetical mean numbers of superior embryos in Hishi and control groups (6 and 4, respectively, with standard deviation of 4) to be 50 patients per group. However, interim assessment conducted at two-thirds of planned trial duration showed that Hishi extract increased cumulative live birth rate considerably more than expected, improving not only embryonic development but also endometrial receptivity. We then ended our trial with 32 enrollees per group, considering the ethical importance of offering potential benefits of Hishi to the control patients in a timely manner.

We decided to report the results of the interim assessment, based on cumulative birth rate, as our final outcome and the primary outcome measure. To elucidate mechanisms of Hishi effects, pre-*vs.*-post treatment changes of serum AGE, hormones, serum biochemical parameters, and OGTT results in the Hishi group were compared with those in controls. FF AGE concentrations and correlation of serum and FF AGE with ART results also were analyzed.

Informed consent was obtained from all patients. The study was approved by the Women’s Clinic Jinno Ethics Committee and registered with the UMIN in Japan (UMIN000017758).

### Ovarian stimulation, IVF, ICSI, and cryopreserved ET

Follicular development was stimulated with the "long protocol," using a GnRH agonist and human menopausal gonadotropin (hMG), as described previously [12]. Briefly, buserelin acetate (Buserecur; Fuji Pharmaceuticals, Tokyo, Japan), at 900 μg per day, was administered nasally beginning on day 18 of the Kaufmann cycle preceding the ART cycle until administration of hCG. Daily administration of 300 IU of hMG (hMG-Ferring; Ferring Pharmaceuticals, Tokyo, Japan) was begun on day 3 of the ART cycle. Human chorionic gonadotropin (HCG-Mochida; Mochida Pharmaceuticals, Tokyo, Japan; 10000 IU) was administered when 1 or more follicles reached a minimum diameter of 18 mm.

Oocytes were collected transvaginally 36 hours after hCG administration and inseminated by motile spermatozoa collected from semen by centrifugation and a swim-up technique as described previously [8]. ICSI was performed when the male partner had severe infertility with a sperm count less than 5 x 10^6^ per mL and/or motility in fewer than 20% of spermatozoa. Oocytes were considered fertilized when 2 pronuclei were observed at 17 to 19 hours after insemination or ICSI. At 2, 3, or 5 days after oocyte retrieval, embryos were transferred to the uterus according to the number and quality of developing embryos for each patient. At 2 and 3 days after oocyte retrieval, morphologically superior embryos were assigned to grade 1 or 2 according to Veeck's criteria [17]. A 25-mg dose of progesterone was administered daily throughout the luteal phase after embryo transfer.

Redundant embryos were cultured to the blastocyst stage and then cryopreserved using a vitrification method. Cryopreserved and thawed blastocysts were transferred to the uterus on luteal day 5 of a spontaneous natural cycle. Ovulation was confirmed by daily examinations by vaginal ultrasonography and serum hormones including E_2_, LH, and progesterone. The day of ovulation, day 0, was defined as the day when a follicle ruptured and progesterone increased above 1.5 ng/mL, following the LH surge before 1 or 2 days. For luteal support, 5000 IU of hCG were administered on luteal days 5, 7, and 9.

### Measurement of AGE

In 29 patients randomized to the Hishi group, various AGE as listed below were measured in serum obtained before and 2 cycles after administration of Hishi extract. Serum for AGE measurement was obtained from 30 control patients following a schedule similar to that for the Hishi group (pre-trial and second intra-trial examinations; Fig. 1). Three Hishi and two control patients were not included in these AGE analyses because 2 Hishi patients and 1 control patient became pregnant using conventional infertility treatment in the first cycle; 1 Hishi patient mis-timed her appointment for the second AGE blood sampling; and 1 control patient stopped coming to the clinic for unknown reasons (dropped out). AGE in FF were measured in 30 Hishi and 29 control patients (Fig. 1). One control patient was not included in FF analyses because her FF was heavily contaminated with blood.

AGE concentrations in samples were measured by electrospray ionization-liquid chromatography-tandem mass spectrometry (ESI-LC-MS/MS) using a TSQ Quantiva triple-stage quadrupole mass spectrometer (Thermo Fisher Scientific, Waltham, MA, USA) as described previously [18]. Standards representing 10 pmol of [^2^H_3_] *N*^δ^-(5-hydro-5-methyl-4-imidazolone-2-yl)-ornithine (MG-H1), [^13^C_6_] *N*^*ω*^-(carboxymethyl)arginine (CMA), [^2^H_2_] *N*^ε^-(carboxymethyl)lysine (CML) (PolyPeptide Laboratories, Strasbourg, France), and 5 nmol of [^13^C_6_] lysine (Cambridge Isotope Laboratories, Tewksbury, MA, USA) were added to the samples. Retention times for MG-H1, CMA, CML and lysine ranged from 12 to 14 min. MG-H1, CMA, CML, lysine, and the standard were detected by electrospray-positive ionization-mass spectrometric multiple-reaction monitoring. Parent ions of MG-H1 and [^2^H_3_] MG-H1 were 229 (*m/z*) and 232 (*m/z*), respectively; fragment ions of 114 (*m/z*) and 117 (*m/z*) from each parent ion were measured for analysis of MG-H1 and [^2^H_3_] MG-H1 in samples. Parent ions of CMA and [^13^C_6_] CMA were 233 (*m/z*) and 239 (*m/z*), respectively; fragment ions of 116 (*m/z*) and 121 (*m/z*) from each parent ion were measured for analysis of CMA and [^13^C_6_] CMA in samples. Parent ions of CML and [^2^H_2_] CML were 205 (*m/z*) and 207 (*m/z*), respectively; fragment ions of 130 (*m/z*) from both parent ions were measured for analysis of CML and [^2^H_2_] CML in samples. As AGE were normalized with respect to lysine content, data are expressed as MG-H1 mmol/mol Lys, CMA mmol/mol Lys and CML mmol/mol Lys [19]. Pentosidine (Pent) concentrations in serum and FF were determined by high performance liquid chromatography (HPLC) using a fluorescent detector as described previously [20]. Quantitative limits measured by authentic standards and coefficients of variation were 5 pmol and 4.4% for MG-H1, 10 pmol and 6.5% for CMA, 1 pmol and 4.0% for CML, and 71.5 fmol and 1.1% for Pent (n=3 in each), respectively.

Serum and FF concentrations of glyceraldehyde-derived AGE (toxic AGE, or TAGE) were measured with a competitive enzyme-linked immunosorbent assay (ELISA) using immunopurified TAGE polyclonal or monoclonal antibody [21]. Results are expressed as TAGE units (U) per mL of serum or FF, with 1 U corresponding to 1 μg of TAGE-bovine serum albumin standard as described previously [21]. Sensitivity and intra- and inter-assay coefficients of variation were 0.01 U/mL, 6.2%, and 8.8%, respectively [8].

### Clinical examinations

After 12 hours of fasting on cycle day 3, all enrolled patients underwent initial clinical examinations (pre-trial examination, Fig. 1), which included determinations of BMI, systolic blood pressure (SBP), and diastolic blood pressure (DBP), a 75-g OGTT, metabolically relevant biochemical analyses, and hormonal measurements. Biochemical tests included glucose metabolism parameters (fasting plasma glucose or FPG; fasting serum insulin or FSI; homeostasis model assessment-insulin resistance index or HOMA-R, defined as FPG x FSI / 405; insulinogenic index or II, defined as [SI at 30 min – FSI] / [PG at 30 min – FPG]; hemoglobin [Hb] A1c; glycoalbumin; and C-peptide), lipid metabolism parameters (total cholesterol or TC, low-density lipoprotein choresterol or LDL, and triglyceride or TG), uric acid (UA), and serum liver enzymes including aspartate aminotransferase (AST), alanine aminotransferase (ALT), γ-glutamyl transpeptidase (γ-GTP), alkaline phosphatase (ALP), lactate dehydrogenase (LDH), and creatine kinase (CK). Hormonal measurements included AMH, follicle-stimulating hormone (FSH), luteinizing hormone (LH), E_2_, prolactin (PRL), testosterone (T), dehydroepiandrosterone sulfate (DHEA-S), thyroid-stimulating hormone (TSH), free thyronine (FT_3_) and free thyroxine (FT_4_).

To assess metabolic and endocrinologic effects of Hishi extract, 18 Hishi and 20 control patients underwent measurements of 6 hormones (FSH, LH, E_2_, PRL, T, and DHEA-S) on cycle day 3, preceding Kaufmann treatment (first intra-trial examination; Fig 1). One cycle later, on ART cycle day 3, those patients repeated the same clinical examinations as initially, excluding hormone measurements (second intra-trial examination; Fig. 1).

Biochemical analyses were performed using routine clinical laboratory methods (LSI Medience, Tokyo, Japan) and serum concentrations of hormones were measured by enzyme chemiluminescent immunoassays.

### Statistical analysis

IBM SPSS Statistics Version 27 (IBM, Tokyo, Japan) was used for all statistical analyses. All data were tested for normality by the Shapiro-Wilk test. If data were not normally distributed, analysis was performed using the Mann-Whitney *U* test, the Wilcoxon matched-pairs signed rank test, or Spearman correlation analyses as appropriate. If data were normally distributed, unpaired *t* tests, paired *t* tests, or Pearson correlation analyses were performed as appropriate. Data also were analyzed using the chi-squared test, Fisher's exact test, or multiple logistic regression analysis as was suitable. P values less than 0.05 were considered to indicate significance. Results are presented as the mean ± standard deviation (SD).

## Results

### Baseline characteristics of patients

No significant differences concerning baseline characteristics were evident between the 2 groups of patients completing the study (Table 1). Among the 32 Hishi patients, 8, 22, 2, and 28 patients respectively had tubal infertility, ovarian dysfunction, endometriosis, and male infertility, as did 5, 21, 1, and 28 of the 31 control patients, showing no significant differences in infertility causes (chi-squared test). Five women in each group were diagnosed with PCOS, representing similar frequency (16% for each group, p = 1.00; Fisher’s exact test). Glucose tolerance was similar between the 2 groups (chi-squared test), including values that were normal, borderline, and diagnostic for diabetes mellitus in 30, 2, and 0 Hishi patients and in 31, 0, and 0 control patients, respectively. Both groups showed normal serum concentrations of hepatic enzymes and thyroid hormones, with no significant differences between the groups.

**Table 1:** Baseline characteristics of patients completing the study ^a^

Characteristic (unit)	Hishi group (n = 32)	Control group (n = 31)
Age (year)	38.9 ± 1.0	39.1 ± 0.9
Infertility duration (years)	2.8 ± 2.9	2.2 ± 2.3
Number of previous ART attempts	0.5 ± 0.7	0.2 ± 0.5
Gravidity	0.9 ± 1.0	1.1 ± 1.6
Parity	0.4 ± 0.6	0.4 ± 0.6
Body mass index (BMI, kg/m^2^)	21.6 ± 2.5	21.0 ± 2.3
Systolic / diastolic blood pressure (mm Hg)	108 ± 14 / 66 ± 8.4	108 ± 11 / 67 ± 7.7
Anti-Müllerian hormone (ng/mL)	2.96 ± 2.36	3.97 ± 4.25
Follicle-stimulating hormone on day 3 (IU/L)	6.8 ± 2.3	7.0 ± 3.0
Luteinizing hormone on day 3 (IU/L)	4.2 ± 1.9	4.9 ± 2.3
Prolactin (ng/mL)	9.8 ± 5.9	11.3 ± 8.9
Estradiol on day 3 (pg/mL)	49 ± 50	79 ± 108
Testosterone on day 3 (ng/mL)	0.19 ± 0.09	0.27 ± 0.43
Fasting plasma glucose (FPG, mg/dL)	81.8 ± 7.7	81.3 ± 7.0
Fasting serum insulin (FSI, μU/mL)	4.5 ± 1.9	4.1 ± 1.3
Hemoglobin A1c (%)	5.3 ± 0.2	5.2 ± 0.2
HOMA-R^b^ (μUg/dL^2^)	0.93 ± 0.44	0.83 ± 0.28
Insulinogenic index (II, dU/g)	1.02 ± 1.62	0.62 ± 3.02
Total cholesterol (TC, mg/dL)	198 ± 32	184 ± 29
Low-density lipoprotein cholesterol (LDL, mg/dL)	112 ± 24	99 ± 33
Triglycerides (TG, mg/dL)	71 ± 51	61 ± 24
Uric acid (UA, mg/dL)	4.3 ± 1.0	4.1 ± 0.7

^a^No significant differences were found between groups (Unpaired *t* test for BMI, systolic / diastolic blood pressure, FPG, FSI, and UA; Mann-Whitney *U* test for other charasteristics)

^b^Homeostasis model assessment-insulin resistance index

In summary, patients who completed the study were endocrinologically and metabolically normal infertile women at ages 38 to 42 years, showing no significant differences between groups in ovarian reserve, potential infertility causes, insulin sensitivity and secretion, or glucose metabolism.

### Clinical outcomes

Sixty-four ART patients were enrolled and randomized to Hishi and control groups (32 patients each). All Hishi patients completed the study; 1 control dropped out for unknown reasons, leaving 31 (Fig. 1). Both groups underwent conventional infertility treatments in the initial cycle, respectively resulting in 2 live deliveries and 1 miscarriage. Two deliveries in the Hishi group were obtained by sexual intercourse, with spontaneous ovulation in one patient and with ovulation following an LH surge induced by GnRHa after rFSH stimulation in the other. In the control patient, intrauterine fetal death occurred at 8 weeks following ovarian stimulation with clomiphene citrate and intrauterine insemination.

**Fig. 2:**
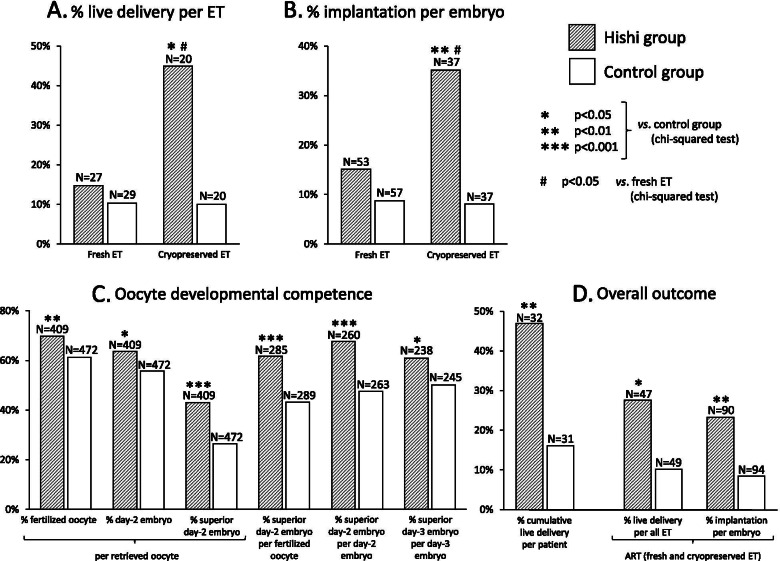
Live delivery rate per embryo transfer (ET) and implantation rate per embryo were compared between Hishi and control groups as well as between fresh and cryopreserved ET (**A** and **B**). Implantation rate per embryo was defined as (number of gestational sacs / number of transferred embryos) x 100%. Oocyte developmental competence (**C**) and overall outcome (**D**) were compared between Hishi and control groups

The remaining 30 patients in each group underwent ovarian stimulation, followed in all by successful oocyte retrieval. Subsequently, 27 and 29 fresh ET were carried out in Hishi and control groups respectively, resulting in 6 and 4 pregnancies with 4 and 3 live deliveries. Pregnancy and delivery rate per fresh ET tended to be higher in the Hishi group (22% and 15%) than in controls (14% and 10%) although statistical significance was not attained (p = 0.50 and p = 0.70, Fisher’s exact test; Fig. 2A). Numbers of transferred embryos were similar between Hishi and control groups (2.0 ± 0.3 and 2.0 ± 0.6, p=0.99, Mann-Whitney *U* test).

Among 17 Hishi and 15 control patients, 20 cycles of cryopreserved ET were carried out in each group, resulting in 9 and 3 pregnancies and 9 and 2 live deliveries, respectively. Hishi extract strikingly increased rates of pregnancies and live deliveries for cryopreserved ET compared with outcomes in controls (45% *vs.* 15%, p<0.05, relative risk [RR] 4.6, 95% confidence interval [CI] 1.02 – 21.0 and 45% *vs.* 10%, p<0.05, RR 7.4, 95% CI 1.3 – 40.5; chi-squared test; Fig. 2A). Numbers of transferred embryos did not differ significantly between Hishi and control groups (1.9 ± 0.4 and 1.9 ± 0.4, p=1.00, Mann-Whitney *U* test). Implantation rate per embryo also was much higher in the Hishi group (35% *vs.* 8.1%, p<0.01, RR 6.1, 95% CI 1.6 – 23.9; chi-squared test; Fig. 2B).

Next we compared fresh and cryopreserved ET within groups. In Hishi patients, implantation rate per embryo was significantly higher in cryopreserved than fresh ET (35% *vs.* 15%, p < 0.05, RR 3.0, 95% CI 1.1 – 8.4; chi-squared test; Fig. 2B). In controls, no significant difference was evident between cryopreserved and fresh ET (8.1% *vs.* 8.8%, p = 1.00; Fisher’s exact test). With Hishi, delivery rate per ET similarly was significantly higher for cryopreserved than fresh ET (45% *vs.* 15%, p < 0.05, RR 4.7, 95% CI 1.2 – 18.7; chi-squared test, Fig. 2A), compared with no significant difference between ET types in controls (10% *vs.* 10%, p = 1.00; Fisher’s exact test). These results suggest that Hishi enhances endometrial receptivity and potential to maintain pregnancy, especially in natural cycles rather than controlled ovarian hyperstimulation (COH).

### Oocyte potential for fertilization and embryonic development

No significant differences were evident between Hishi and control groups (n = 30 in each) in number of follicles larger than 16 mm in diameter (6.5 ± 3.7 and 6.7 ± 3.6, respectively), serum E_2_ (3410 ± 2470 pg/mL and 3470 ± 2020), or endometrial thickness (11.2 ± 2.0 mm and 10.8 ± 3.6) on the day of hCG administration (Mann-Whitney *U* test). Numbers of retrieved oocytes (13.6 ± 10.4 and 15.7 ± 9.4) and mature oocytes (6.1 ± 6.2 and 5.9 ± 4.4) did not differ significantly between Hishi and control groups (Mann-Whitney *U* test).

Developmental potentials of oocytes and embryos, however, were significantly greater in the Hishi group (Fig. 2C). Oocytes were significantly more likely to develop into fertilized oocytes, day-2 embryos, or superior day-2 embryos in the Hishi group than in controls. The Hishi group also showed a significantly higher rate of fertilized oocytes developing into superior day-2 embryos. Further, significantly more day-2 embryos as well as day-3 embryos showed favorable morphology in the Hishi group.

Embryo transfers were carried out at 2, 3, and 5 days after oocyte retrieval in 2, 12, and 13 Hishi subjects and in 3, 8, and18 control subjects, respectively. The distribution of ET days did not differ significantly between the 2 groups (chi-squared test).

Data from IVF and ICSI were analyzed together since no significant differences were present (Mann-Whitney *U* test) in the number of fertilized oocytes (9.6 ± 8.4 *vs.* 7.6 ± 5.9, respectively) or day-2 embryos (8.8 ± 8.0 *vs.* 6.4 ± 6.0) between IVF and ICSI. Further, delivery rates did not differ significantly among IVF, ICSI, and IVF+ICSI patients (9.8, 9.1, and 25%, respectively; chi-squared test).

### Overall study outcomes

In 32 Hishi *vs.* 31 control patients completing the study, 15 *vs.* 5 live deliveries were achieved by either conventional infertility treatments (2 *vs.* 0 deliveries) or ART (13 *vs.* 5). Thus, cumulative frequency of live delivery per patient was 47% in the Hishi group, significantly higher than 16% in controls (p<0.01, RR 4.6, 95% CI 1.4 – 15.0, chi-squared test; Fig. 2D). In ART including both fresh and cryopreserved ETs, live delivery rate per ET and implantation rate per embryo both were significantly higher in the Hishi group than in controls (28% *vs.* 10%, p<0.05, RR 3.4, 95% CI 1.1 – 10.4 and 23% *vs.* 8.5%, p<0.01, RR 3.3, 95% CI 1.4 – 7.8; chi-squared test; Fig. 2D).

Associations of 4 major fertility-related factors (age, day-3 FSH, AMH, and Hishi) with achievement of cumulative live delivery were analyzed by logistic regression analysis. Among these factors, only Hishi significantly correlated with cumulative live delivery (p < 0.05, odds ratio 5.1, 95% CI 1.4 – 18.3).

Fifteen Hishi and five control patients respectively delivered 17 (8 male, 9 female) and 5 (2 male, 3 female) normal live infants, including 2 and 0 sets of twins. Considering the 13 and 5 singleton newborns in Hishi and control groups, no significant difference was evident in body weight (2845 ± 723 *vs.* 3023 ± 108 g; unpaired *t* test) or gestational age at delivery (37.5 ± 3.0 *vs.* 38.0 ± 1.2 weeks; Mann-Whitney *U* test).

No adverse events or side effects from Hishi were observed among Hishi group patients throughout the study period, nor in their fetuses or infants.

### Effect of Hishi on AGE

**Fig. 3:**
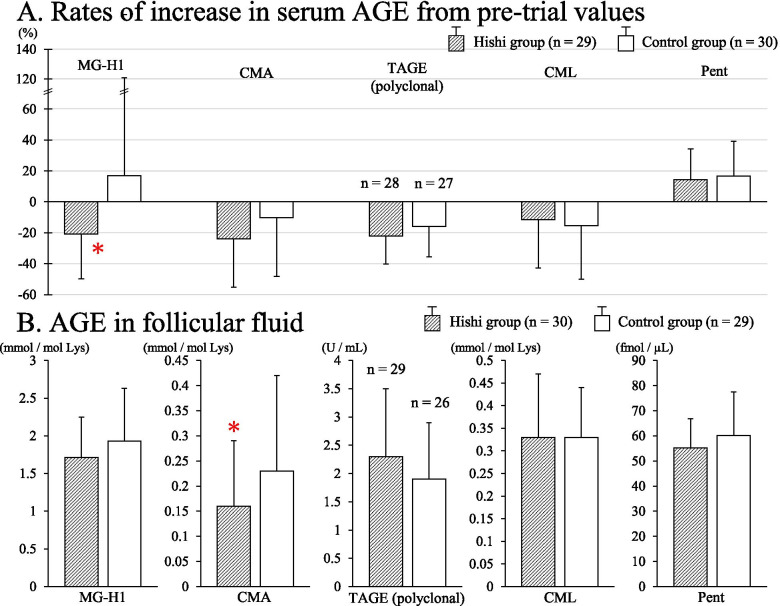
Rates of increase for serum AGE from the pre-trial values (**A**) and follicular fluid AGE (**B**) in Hishi and control groups. The increase rate was defined as [(intra-trial value minus pre-trial value) / pre-trial value] x 100%. * P < 0.05 *vs.* control group, Mann-Whitney *U* test

Serum concentrations of *N*^*ω*^-(carboxymethyl) arginine (CMA) and *N*^δ^-(5-hydro-5-methyl-4-imidazolone-2-yl)-ornithine (MG-H1) decreased significantly between pre-trial and intra-trial determinations in the Hishi group (n = 29: CMA, from 0.73 ± 0.19 mmol/mol Lys to 0.53 ± 0.16, p < 0.001; paired *t* test; and MG-H1, from 4.78 ± 1.86 mmol/mol Lys to 3.55 ± 1.25, p < 0.001; paired *t* test). Significant decreases did not occur in controls (n = 30: CMA, from 0.68 ± 0.17 to 0.59 ± 0.26, p = 0.09, Wilcoxon matched-pairs signed rank test; and MG-H1, from 3.41 ± 1.48 to 3.26 ± 1.79, p = 0.4; Wilcoxon matched-pairs signed rank test). The rate of increase for MG-H1, defined as [(intra-trial value minus pre-trial value) / pre-trial value] x 100%, was significantly lower in the Hishi group than in controls (-20.8 ± 28.9%, n = 29 *vs.* 16.9 ± 104.2, n = 30; p < 0.05, Mann-Whitney *U* test; Fig. 3A), meaning that MG-H1 decreased significantly more in Hishi patients. Concentrations of CMA in FF were significantly lower in Hishi patients than controls (0.16 ± 0.13 mmol/mol Lys, n = 30 *vs.* 0.23 ± 0.19, n = 29; p < 0.05, Mann-Whitney *U* test; Fig. 3B). Thus, Hishi decreased serum CMA and MG-H1, as well as FF CMA.

Serum *N*^ε^-(carboxymethyl)lysine (CML) and toxic AGE (TAGE) decreased and serum pentosidine (Pent) increased significantly in both Hishi (n = 29) and control (n = 30) groups (CML, from 0.45 ± 0.18 mmol/mol Lys to 0.38 ± 0.15, p < 0.05 in the Hishi group and from 0.50 ± 0.21 to 0.41 ± 0.23, p < 0.01 in the control group, Wilcoxon matched-pairs signed rank test; TAGE, from 10.7 ± 3.0 U/mL, n = 28, to 8.3 ± 2.8, n = 28, p < 0.001, paired *t* test in the Hishi group and from 9.3 ± 3.2, n = 27, to 7.6 ± 2.3, n = 27, p < 0.001, Wilcoxon matched-pairs signed rank test in the control group; and Pent, from 82.4 ± 17.2 fmol/μL to 92.2 ± 15.6, p < 0.01, paired *t* test in the Hishi group and from 88.3 ± 23.2 to 101.5 ± 29.3, p < 0.01, Wilcoxon matched-pairs signed rank test in the control group). No significant differences were detected between groups in rates of increase for CML, TAGE, or Pent as well as their concentrations in FF (Mann-Whitney *U* test for CML; unpaired *t* test for TAGE and Pent; Fig. 3). Thus, changes in serum CML, TAGE, and Pent observed in both groups may reflect shared interventions such as life-style modification and suppression of ovulation by the Kaufmann cycle.

**Fig. 4:**
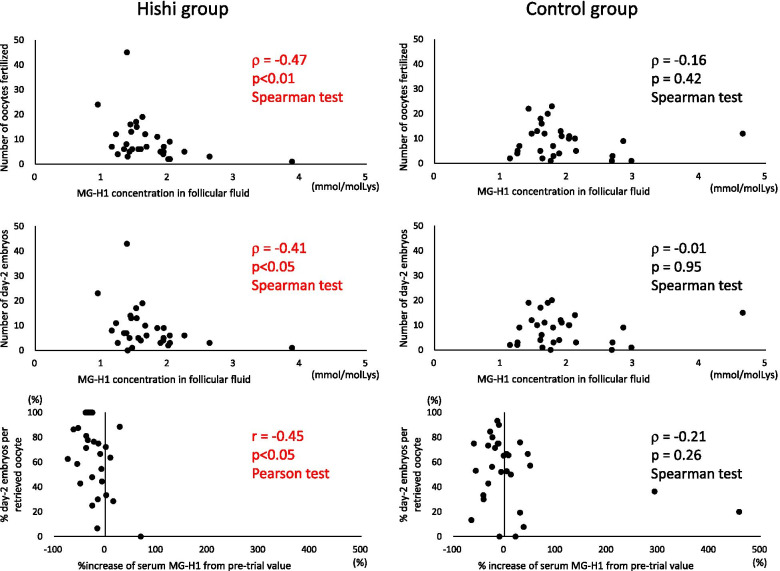
Scatter plots of some correlations presented in Table 2. Dots tend to lie within the left upper quadrant in the Hishi group more than in the control group

MG-H1 concentrations in FF showed a significant negative correlation with numbers of follicles measuring 12 mm or larger on the hCG day, oocytes retrieved, oocytes fertilized, and day-2 embryos in the Hishi group but not controls (Table 2, Fig. 4). In other words, follicular development and oocyte quality improved in association with decreasing FF MG-H1 concentrations in Hishi patients, but no such relationship was seen in controls. Before Hishi was given, follicular development presumably was similar between groups irrespective of MG-H1 concentrations in FF, considering that group assignment of patients was random. Hishi likely improved follicular development and oocyte quality by decreasing AGE in ovarian follicles, especially in patients showing larger reductions.

**Table 2:** Correlation of MG-H1 with follicular development, fertilization, embryonic development, and implantation in Hishi and control groups ^a^

	Hishi group		Control group	
	r (n)	p-value	r (n)	p-value
MG-H1 concentration in follicular fluid …				
*vs.* number of follicles ≧12 mm on hCG day	-0.38 (30)	<0.05	-0.24 (29)	0.20
*vs.* number of oocytes retrieved	-0.46 (30)	<0.05	-0.22 (29)	0.26
*vs.* number of oocytes fertilized	-0.47 (30)	<0.01	-0.16 (29)	0.42
*vs.* number of day-2 embryos	-0.41 (30)	<0.05	-0.01 (29)	0.95
% increase of serum MG-H1 from pre-trial value				
*vs.* % day-2 embryos per retrieved oocyte	-0.45 (29)	<0.05	-0.21 (30)	0.26
Pre-trial serum MG-H1 concentration …				
*vs.* % superior day-2 embryos per day-2 embryo	0.37 (30)	<0.05	-0.01 (30)	0.97
*vs.* endometrial thickness	0.11 (29)	0.56	-0.51 (30)	<0.01

^a^ Correlations between % increase of serum MG-H1 and % day-2 embryos per retrieved oocyte, and between pre-trial serum MG-H1 and endometrial thickness in the Hishi group, were analyzed by the Pearson test; all other correlations were analyzed by the Spearman test

Increases in serum MG-H1 from pre-trial values correlated negatively with development of retrieved oocytes to day-2 embryos in the Hishi group but not controls (Table 2, Fig. 4); the more serum MG-H1 decreased with Hishi, the more likely oocytes developed to day-2 embryos. Pre-trial serum MG-H1 concentrations correlated positively with percentage of superior day-2 embryos among all day-2 embryos in the Hishi group but not controls, suggesting that Hishi is particularly effective in improving embryo quality in patients with elevated AGE at beginning of treatment. Further, pre-trial serum concentrations of MG-H1 showed a significant negative correlation with endometrial thickness in controls but not the Hishi group (Table 2), suggesting that Hishi improved endometrial conditions by decreasing AGE. Also, pre-trial serum TAGE concentrations showed a significant positive correlation with implantation rates per freshly transferred embryo in the Hishi group (ρ = 0.40, n = 26, p < 0.05, Spearman test) but not controls (ρ = -0.08, n = 26, p = 0.71, Spearman test), suggesting that Hishi may be particularly effective in improving endometrial receptivity in patients with elevated TAGE concentrations.

### Influence of Hishi extract on clinical examination and routine laboratory findings

No significant differences were evident between Hishi and control groups in pre-trial, first intra-trial, and second intra-trial examinations, including BMI, SBP, DBP, OGTT, various biochemical analyses, and hormone measurements--except for TC, which decreased significantly in the control group (from 189 ± 33 mg/dL, n = 20, to 176 ± 20, n = 20, p < 0.05, paired *t* test) but not in Hishi patients (initially 196 ± 21 mg/dL, n = 18, and later 191 ± 21, n = 18, p = 0.39; paired *t* test). This resulted in a significantly lower intra-trial TC value in controls than in the Hishi group (p < 0.05, unpaired *t* test).

## Discussion

To our knowledge, this report presents the first successful infertility treatment involving AGE reduction. Hishi extract significantly decreased serum and FF AGE, greatly increasing live birth with ART for women approximately 40 years old. Benefits associated with Hishi included better oocyte quality and, especially in natural cycles, endometrial conditions. These improvements likely resulted from AGE reduction in ovarian follicles and endometrium. Decreases in arginine-derived AGEs such as MG-H1 and CMA were associated with improved follicular state.

We previously linked increased TAGE, Pent, and CML in FF and TAGE in serum with poorer follicular growth, likelihood of fertilization, and embryonic development, while elevated serum TAGE and FF Pent decreased likelihood of successful pregnancy [8]. These observations were supported by two recent reports: FF AGE concentration was negatively associated with numbers of oocytes retrieved, oocytes fertilized, and high-quality embryos [22], while FF AGE and the AGE/soluble RAGE (sRAGE) ratio were significantly higher in follicles containing oocytes that developed into morphologically poor embryos [23]. On the other hand, FF concentrations of sRAGE, which counteracts AGE effects, correlated positively with numbers of retrieved oocytes, fertilized oocytes, high-quality embryos, and likelihood of pregnancy [9, 24, 25].

Accumulation of AGE in tissues and cells induces macromolecular trapping and cross-linking, causing molecules to malfunction and resist removal by proteolysis [1, 5]. Directly, and indirectly through RAGE, AGE increases oxidative stress [1], a major cause of macromolecular damage in follicular [26] and other cells [4] during aging. Accumulation of AGE in follicles was shown to reduce oocyte competence by triggering inflammation via activation of ATF4 in the follicular microenvironment [23]. In PCOS, the AGE-RAGE system causes decreases in glucose uptake and Glut-4 expression by granulosa cells, abnormalities of follicular extracellular matrix organization, and interference with LH action in granulosa cells through activation of the ERK 1/2 pathway [9]. All of these AGE effects are likely to impair folliculogenesis, although underlying mechanisms require further clarification.

We therefore set out to improve ART outcome by AGE reduction using Hishi extract, which was shown *in vitro* to inhibit production of AGE and their intermediates more strongly than aminoguanidine and to disrupt α-dicarbonyl compounds and AGE crosslinks more effectively than *N*-phenacylthiazolium bromide, a dicarbonyl link-breaker [14].

In our study, Hishi extract decreased certain serum and FF AGE, specifically MG-H1 and CMA, more than TAGE, Pent, and CML; the latter AGE were significantly associated with ART outcome in our previous report [8]. While MG-H1 and CMA are most sensitive to *in vivo* effects of Hishi, they do not necessarily dominate pathogenic processes compromising follicular function. Glycation/carbonyl stress generates various AGE *in vivo*, including less harmful AGE without direct cytotoxic effects such as CML, Pent, MG-H1, and pyrraline, as well as toxic, highly pathogenic AGE such as TAGE [3]. Some non-toxic AGE may be components of biologic defense mechanisms working against accumulation of toxic AGE [3]. MG-H1 and CMA were found to be elevated in diabetic patients [27, 28], but implications for pathogenesis in diabetes remain unclear. However, concentrations of even non-toxic AGE serve as indicators of trends in overall glycation/carbonyl stress, such as decreased concentrations of MG-H1 correlating with improved functional assessments of follicular development, fertilization, embryonic development, and implantation in the present study.

Our previous study [8] involved consecutive ART patients with no limitation of age, which ranged from 25 to 45 years. In contrast, the present study included only patients between 38 and 42 years old. In the previous study population, age, FF-Pent, and serum TAGE each affected the likelihood of ongoing pregnancy [8]. We believe that patient age differences could account for the differences in results concerning AGE. Assay methods also differed between these studies; CML and Pent respectively were measured by ESI-LC-MS/MS and HPLC in the present investigation, while both were measured by enzyme-linked immunosorbent assay in the previous report.

Among our present observations concerning AGE, particularly important points are that Hishi decreased AGE *in vivo* while reduction of AGE correlated with improved folliculogenesis, fertilization, embryonic development, and implantation, which suggests AGE decreases as a therapeutic mechanism likely to underlie fertility enhancement by Hishi.

How Hishi decreases AGE *in vivo* is much less clear. Polyphenols from water chestnut (*Trapa japonica*) husk were found to inhibit α-amylase and α-glucosidase, reducing blood glucose and insulin concentrations in mice [29]. The present study, however, showed little effect of Hishi extract on such glucose metabolism metrics as fasting plasma glucose, serum insulin, HOMA-R, insulinogenic index, hemoglobin A1c, glycoalbumin, C-peptide, and OGTT. The discrepancy may reflect differences between animal and human studies in dosage (40 mg/kg in mice *vs.* 2 mg/kg in our study) or species-specific factors. According to our study, Hishi extract appeared unlikely to decrease AGE indirectly by reductions in blood glucose or enhanced insulin sensitivity or secretion. More likely, Hishi extract directly inhibited production of AGE and their intermediates or disrupted their bridging structures, as has been demonstrated *in vitro* [14]. In our present study, MG-H1 and CMA, which are readily generated *in vitro* from the α-dicarbonyl compounds, methylglyoxal [19] and glyoxal, respectively [18], were particularly sensitive to the effects of Hishi, which inhibits production of α-dicarbonyl compounds *in vitro* while also degrading them [14].

In Hishi patients, implantation enhancement was more evident for cryopreserved than fresh ET (35% *vs.* 15%). Improved implantation reflected increased endometrial receptivity more than embryo quality, considering that both ET strategies involved sibling embryos from the same oocyte retrieval. Endometrial receptivity is often better in natural than in COH cycles [30]. However, no significant difference in implantation rate was observed between cryopreserved and fresh ET among controls (8.1% *vs.* 8.8%), arguing against an advantage of natural cycles as the reason. The simplest explanation would be that Hishi improves endometrial receptivity but COH somehow interferes with this effect. Ability of Hishi extract to improve endometrial receptivity likely is related to lowering of AGE. This mechanism is supported by a negative correlation between pre-trial serum MG-H1 and endometrial thickness in controls, while pre-trial serum TAGE showed significant positive correlation with implantation rates in Hishi patients.

Previous reports also suggest that AGE can impair implantation and placental function. Glucose-derived AGE induced secretion of chemokines and apoptosis in human first-trimester trophoblasts *in vitro* [31]. Obese women were found to have elevated AGE, RAGE, and inflammatory cytokines in endometrial fluid and tissue, while increased concentrations of AGE inhibited trophectodermal adhesion and trophoblast invasion *in vitro* [32]. Treatment during pregnancy with metformin, an insulin sensitizer known to decrease AGE [1], was found to reduce risk of late pregnancy loss and preterm birth in women with PCOS [33].

Endometrium is shed during menstruation and regenerates during the next cycle, while an ovarian follicle grows from a primordial follicle until ovulation occurs after about 6 months, then becoming atretic. Endometrial dysfunction differs from most IR-related conditions such as diabetes, hypertension, and metabolic syndrome, where affected tissues are not renewed. Cyclic renewal of endometrium may facilitate clinical interventions to control endometrial AGE accumulation, making benefits of AGE reduction by Hishi more evident for endometrial receptivity than for other health issues affected by IR [1], where earlier and more sustained AGE reduction by Hishi or other means might be required for clinical benefit. Hishi may be particularly suited to long-term administration if needed, considering its history of safety [13].

Hishi extract showed no side effects or adverse events in our Hishi group patients or their fetuses during the study period. All infants in the Hishi group were free of congenital anomalies and had body weights similar to those in the control group. Hishi extract is a spray-dried powder prepared from a solution extracted by heating dried and crushed water chestnut rinds with water. This method of production is fundamentally the same as for traditional Hishi tea, long consumed in the north Kyushu area of Japan [34]; that region has shown no known excesses of obstetric problems or congenital anomalies compared with other parts of Japan [35]. Safety of Hishi extract was also demonstrated by a mutation toxicity test in mice, a reproductive development/toxicity screening test in rats, and an overdose safety study in humans [36]. Thus, Hishi extract likely would be safe for would-be expectant mothers, fetuses, and children with exposure *in utero*. Further confirmation of safety of Hishi extract is desirable

After an interim assessment of results showed stronger evidence for benefit from Hishi than expected, we stopped our study early in order to offer potential clinical benefits of Hishi to our control group. This decision decreased numbers of patients available for comparisons, representing a limitation of this study. We also excluded women with severely diminished ovarian reserve from this study, so efficacy of Hishi extract in such patients awaits further investigation. In this study, an envelope method was used for randomization. Although such randomization might increase risk of bias to some extent, it represents a reasonable method for a small RCT. We minimized the risk of bias by strictly controlling the envelope drawing, permitting no redraws.

The present study demonstrated successful treatment of infertility by AGE reduction, a new approach to infertility treatment. This advance involving Hishi also might spur new clinical trials of AGE reduction using Hishi or other agents against other AGE-associated disorders such as diabetes and hypertension, while avoiding the toxicity issues previously encountered with aminoguanidine. Meaningful assessment in diabetes or hypertension may require earlier and more sustained reduction of AGE; Hishi extract should be particularly suited to relatively long assessments, considering its long history of safety.

## Conclusions

This report presents the first successful randomized clinical trial of infertility treatment involving AGE reduction. Administration of Hishi extract significantly decreased serum and FF AGE, enhanced oocyte developmental potential, and improved endometrial receptivity, especially in natural cycles. Consequently, Hishi extract greatly increased the cumulative live birth rate and live birth rate per ET in ART patients approximately 40 years old. These improvements likely resulted from AGE reduction in ovarian follicles and endometrium. Decreases in arginine-derived AGEs such as MG-H1 and CMA were associated with improved follicular state. Successful treatment of infertility by AGE reduction with Hishi extract represents an important new addition to infertility treatment. Assessment of Hishi as a supplemental aid in management of other AGE-associated conditions such as diabetes and hypertension may be warranted.

## Data Availability

The datasets used and/or analysed during the current study are available from the corresponding author on reasonable request.

## Abbreviations

AGE: Advanced glycation end-products
AMH: Anti-Müllerian hormone
ART: Assisted reproductive technology
BMI: Body mass index
CI: Confidence interval
CMA: *N*^*ω*^-(carboxymethyl)arginine
CML: *N*^ε^-(carboxymethyl)lysine
DHEA-S: Dehydroepiandrosterone sulfate
COH: Controlled ovarian hyperstimulation
DBP: Diastolic blood pressure
ET: Embryo transfer
E_2_: 17β-estradiol
FPG: Fasting plasma glucose
FSI: Fasting serum insulin
FF: Follicular fluid
GnRH: Gonadotropin-releasing hormone
FSH: Follicle-stimulating hormone
FT_3_: Free thyronine
FT_4_: Free thyroxine
Hb: Hemoglobin
HOMA-R: Homeostasis model assessment-insulin resistance index
hCG: Human chorionic gonadotropin
hMG: Human menopausal gonadotropin
II: Insulinogenic index
IR: Insulin resistance
ICSI: Intracytoplasmic sperm injection
IVF: *in vitro* fertilization
LDL: Low-density lipoprotein choresterol
LH: Luteinizing hormone
MG-H1: *N*^δ^-(5-hydro-5-methyl-4-imidazolone-2-yl)-ornithine
OGTT: 75-g oral glucose tolerance test
OPU: Oocyte pick-up
OHSS: Ovarian hyperstimulation syndrome
OS: Ovarian stimulation
Pent: Pentosidine
PCOS: Polycystic ovary syndrome
PRL: Prolactin
RAGE: Receptor for AGE
RR: Relative risk
SD: Standard deviation
SBP: Systolic blood pressure
T: Testosterone
TC: Total cholesterol
TG: Triglyceride
TAGE: Toxic AGE
TSH: Thyroid-stimulating hormone
UA: Uric acid
